# Functional Characterization of the Soybean *Glycine max* Actin Depolymerization Factor GmADF13 for Plant Resistance to Drought Stress

**DOI:** 10.3390/plants13121651

**Published:** 2024-06-14

**Authors:** Deying Wang, Mengxue Du, Peng Lyu, Jingyu Li, Huiran Meng, Xinxin Liu, Mengmeng Shi, Yujie Gong, Qi Sha, Qingmei Men, Xiaofei Li, Yongwang Sun, Shangjing Guo

**Affiliations:** 1 School of Agricultural Science and Engineering, Liaocheng University, Liaocheng 252059, China; wdy1999122@foxmail.com (D.W.); dumengxue1220@163.com (M.D.); m17663279431@163.com (P.L.); lijy0801@163.com (J.L.); meng001004@163.com (H.M.); liuxinxin0518123@163.com (X.L.); shimengmeng2022@163.com (M.S.); 18753340145@163.com (Y.G.); m18764495501@163.com (Q.S.); 15163365595@163.com (Q.M.); lixiaofei9907@163.com (X.L.); 2 College of Life Sciences, Qingdao Agricultural University, Qingdao 266109, China

**Keywords:** soybean (*Glycine max*), drought stress, *GmADF13*, actin-depolymerizing factor

## Abstract

Abiotic stress significantly affects plant growth and has devastating effects on crop production. Drought stress is one of the main abiotic stressors. Actin is a major component of the cytoskeleton, and actin-depolymerizing factors (ADFs) are conserved actin-binding proteins in eukaryotes that play critical roles in plant responses to various stresses. In this study, we found that *GmADF13*, an *ADF* gene from the soybean *Glycine max*, showed drastic upregulation under drought stress. Subcellular localization experiments in tobacco epidermal cells and tobacco protoplasts showed that GmADF13 was localized in the nucleus and cytoplasm. We characterized its biological function in transgenic *Arabidopsis* and hairy root composite soybean plants. *Arabidopsis* plants transformed with *GmADF13* displayed a more robust drought tolerance than wild-type plants, including having a higher seed germination rate, longer roots, and healthy leaves under drought conditions. Similarly, *GmADF13*-overexpressing (OE) soybean plants generated via the *Agrobacterium rhizogenes*-mediated transformation of the hairy roots showed an improved drought tolerance. Leaves from OE plants showed higher relative water, chlorophyll, and proline contents, had a higher antioxidant enzyme activity, and had decreased malondialdehyde, hydrogen peroxide, and superoxide anion levels compared to those of control plants. Furthermore, under drought stress, *GmADF13* OE activated the transcription of several drought-stress-related genes, such as *GmbZIP1*, *GmDREB1A*, *GmDREB2*, *GmWRKY13*, and *GmANK114*. Thus, *GmADF13* is a positive regulator of the drought stress response, and it may play an essential role in plant growth under drought stress conditions. These results provide new insights into the functional elucidation of soybean *ADFs*. They may be helpful for breeding new soybean cultivars with a strong drought tolerance and further understanding how *ADFs* help plants adapt to abiotic stress.

## 1. Introduction

Plants, as sessile organisms, are often subjected to abiotic stresses that significantly affect their growth and development [1]. Abiotic stresses refer to any non-living factors in specific environments that adversely impact the normal growth and development of plants, leading to disruptions in cellular metabolism, gene regulation, and other biological processes [2,3]. Specifically, abiotic stresses primarily include drought, soil salinity, and extreme temperature fluctuations, among others. Different stresses result in different damages, all of which ultimately impair plant productivity [2,4]. Drought stress, one of the major abiotic stress factors, exerts significant impacts on plant growth and development at physiological, biochemical, and molecular levels [5]. It poses a major threat to agriculture worldwide, hindering food security [6,7]. Therefore, the development of stress-tolerant crops with stable yields under adverse conditions is of great importance [8,9].

Actin is mainly found in cells as globular actin (G-actin) and filamentous actin (F-actin). F-actin is the primary form of actin that mediates biological functions and constitutes the major component of the plant cell cytoskeleton [10]. Its polymerization, depolymerization, crosslinking, and bundling processes are regulated by a series of actin-binding proteins (ABPs) [11,12,13]. Among them, actin depolymerization factors (ADFs) are one of the most essential ABPs, and they depolymerize or sever actin filaments to promote microfilament reorganization [14,15]. ADFs are characterized by their small size (15–22 kDa) and high conservation, and they are ubiquitous in all eukaryotic cells [16,17]. The first ADF was isolated from the chicken embryo brain [18], and ADFs were subsequently found in many other eukaryotes [19]. Plant ADFs are involved in various biological processes [20], such as cell growth [21], flower development [22], nodule organogenesis in leguminous crops [23], the growth of the tip of elongating root hairs [24], and pollen tube growth [25]. Additionally, *ADF* genes participate widely in plant responses to various biotic and abiotic stresses [26]. For example, *AtADF2* positively regulates plant resistance to root-knot nematodes [27], and *TaADF3* [28], *TaADF4* [29], and *TaADF7* [30] modulate wheat resistance to stripe rust (*Puccinia striiformis*). A recent study showed a significant correlation between *ZmADF4* and resistance to the Mediterranean corn borer (*Sesamia nonagrioides*) [31]. Furthermore, *DaADF3*, an *ADF* gene from Antarctic hair grass (*Deschampsia antarctica*), plays a vital role in the enhancement of cold tolerance in transgenic rice plants and the adaptation of Antarctic hair grass to its extreme environment [32]. The overexpression of *AtADF1* enhances the ability of *Arabidopsis thaliana* to resist salt stress by the AtMYB73-mediated salt stress response pathway [33]. The heterologous overexpression of *OsADF3* improves the drought tolerance of *Arabidopsis* [34]. Drought stress can induce the expression of *AtADF5* in *Arabidopsis* [35]. Compared to a wild-type (WT) plant, the *Atadf5* mutant exhibits a reduced formation of cell microfilament bundles under drought conditions, delayed stomatal closure, exacerbated leaf dehydration, and decreased survival rates. A further investigation revealed that *AtADF5* regulates stomatal movement by affecting the rearrangement of actin filaments, thereby enhancing plant adaptation to drought stress [35]. The overexpression of *ZmADF5* in maize (*Zea mays*) and *Arabidopsis* reduces stomatal aperture, decreases the water loss rate, enhances the cellular scavenging of reactive oxygen species (ROS), and improves drought tolerance in transgenic plants [36]. The association of these ADF functions with abiotic stress suggests their crucial role in plants under unfavorable conditions.

The soybean (*Glycine max*) is an economically important legume crop grown worldwide as a source of edible oil, vegetative protein, and biodiesel [37,38,39]. Soybean is an important crop, but various stresses threaten its production. Among these, drought stress is one of the most important abiotic stresses, and it can seriously affect the growth and yield of soybeans [40,41]. Therefore, to promote soybean production and crop security on less-than-ideal land, the drought tolerance of soybeans must be enhanced [42,43]. The physiological and molecular mechanisms of soybean resistance to drought should be investigated to improve its resistance by discovering drought-resistant genes [44].

Many genes related to drought resistance in soybeans have been reported. For example, GmNAC8, an NAC (NAM, ATAF1/2, CUC1/2) protein, enhances drought tolerance in soybeans by interacting with the GmDi19-3 protein [45]. Molecular studies have revealed that GmMYB14 (myeloblastosis) mediates brassinosteroid signaling pathways to enhance the drought tolerance of soybeans [46]. The interaction between GmNTF2B-1 (nuclear transport factor 2B family) and GmOXR17 (oxidoreductase) might enhance GmOXR17’s nuclear import capacity, thereby boosting its nuclear ROS clearance ability and improving its soybean drought resistance [47]. Further research on drought resistance genes in soybeans can enrich our understanding of the underlying molecular mechanisms and provide a basis for breeding drought-resistant varieties. 

Our previous research indicated the widespread expression of *GmADFs* in various organs and tissues, with most responding actively to abiotic stress, suggesting that they play critical roles in various biological processes [48]. Among these genes, the expression level of *GmADF13* (Gene ID: *Glyma.12G031700* in the phytozome database; the gene name is defined in [48]) was drastically upregulated under drought stress, indicating that it may be involved in the plant response to drought stress [48]. In this study, we investigated the drought stress tolerance conferred by *GmADF13* in both *Arabidopsis* and soybeans, and the results showed that the overexpression of *GmADF13* enhanced the ability of transgenic plants to resist drought stress. Identifying the function of GmADF13 will contribute to a better understanding of how ADF proteins aid soybeans in adapting to abiotic stress and uncovering potential mechanisms of the actin cytoskeleton in response to drought stress. 

## 2. Results

### 2.1. Cloning and Sequence Analysis of GmADF13

**Figure 1:**
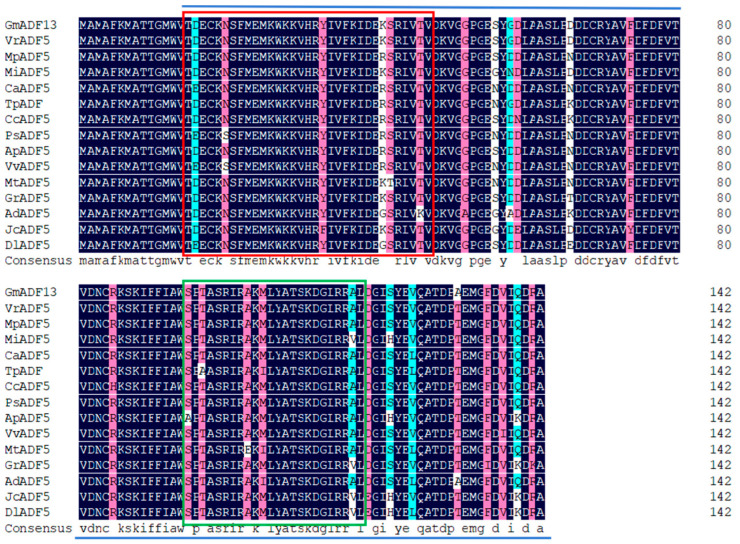
Alignment of the putative amino acid sequence of GmADF13 with that of other plant ADF proteins. Identical amino acids are shaded in black. The putative actin-binding region, CAM binding site, and ADF-H domain are enclosed by the green box, red box, and blue line, respectively. The black, pink, and blue shading represent amino acid similarities of 100%, >75%, and >50%, respectively.

A PCR amplification was performed using the cDNA of young soybean sprouts as a template using GmADF13-specific primers (Supplementary Table S1). The sequencing showed that the coding sequence (CDS) of *GmADF13* was 432 bp long, with a genomic sequence of 1377 bp. Fourteen ADF proteins from different plant species were used for the alignment of multiple sequences (Figure 1) (Supplementary Table S2) to identify the conservation of ADF proteins. We found that GmADF13 is highly homologous to other ADF proteins in plants. A structural analysis revealed that the ADF homology (ADF-H) conserved domain was found at amino acid positions 15–142 in CmADF13. The CAM binding site was found at positions 15–47, and the actin-binding region was found at positions 95–128. Among them, the ADF-H conserved domain is the hallmark domain of the ADF family and is highly conserved among different species. 

**Figure 2:**
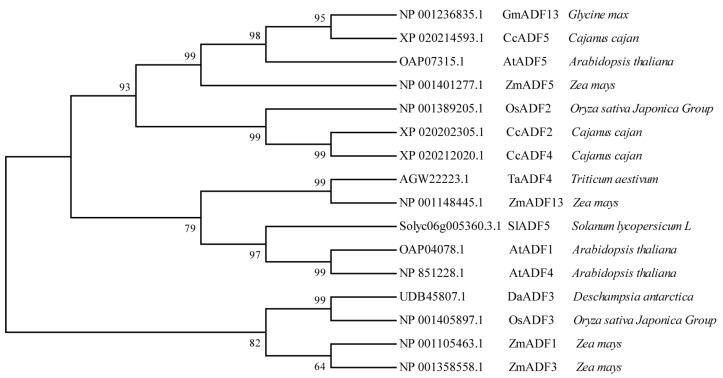
Phylogenetic analysis of GmADF13 and closely related ADFs from other species. MEGA 11.0 software was employed to construct phylogenetic trees using the neighbor-joining method. The parameters utilized were the Poisson model, pairwise deletion, and 1000 bootstrap replications.

We selected 15 reported ADF proteins and used the neighbor-joining method in MEGA 11.0 to construct a phylogenetic tree to verify their evolutionary relationship with GmADF13 (Figure 2) (Supplementary Table S3). The results showed that the GmADF13 protein formed a large branch with the CcADF5, AtADF5, and ZmADF5 proteins, indicating that they have high homology.

### 2.2. GmADF13 Encodes Nucleus- and Cytoplasm-Localized ADF Proteins

The localization of a protein is usually related to its function. To analyze the subcellular localization of GmADF13, its full-length cDNA without the termination codon was fused to the green fluorescent protein (GFP) reporter gene under the control of the 35S promoter. The recombinant construct and the GFP vector were independently introduced into tobacco (*Nicotiana benthamiana*) epidermal cells and tobacco protoplasts, respectively. 

**Figure 3:**
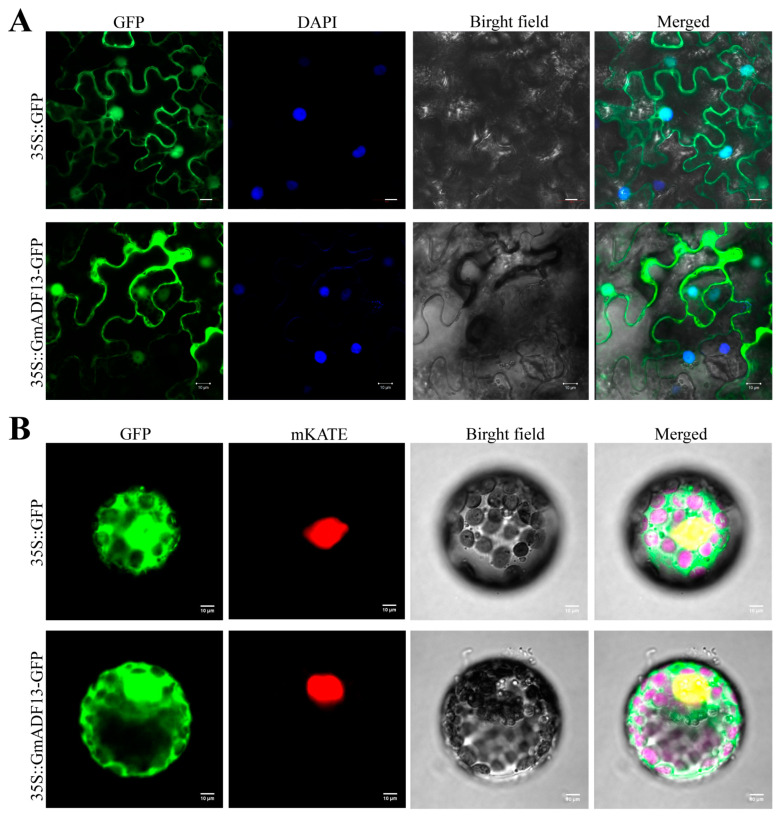
Subcellular localization of GmADF13. (**A**) Subcellular localization of GmADF13 in tobacco epidermal cells. (**B**) Subcellular localization of GmADF13 in tobacco protoplasts. Scale bar = 10 µm. GmADF13-GFP fusion protein localized in the nucleus and cytoplasm.

A confocal scanning analysis showed that the 35S::*GmADF13*-GFP fusion protein localized in the nucleus and cytoplasm; conversely, the 35s::GFP fluorescence signal was distributed throughout the cell. This confirmed that GmADF13 is localized in the nucleus and cytoplasm (Figure 3A,B).

### 2.3. Arabidopsis Overexpressing GmADF13 Exhibited Enhanced Drought Tolerance

**Figure 4:**
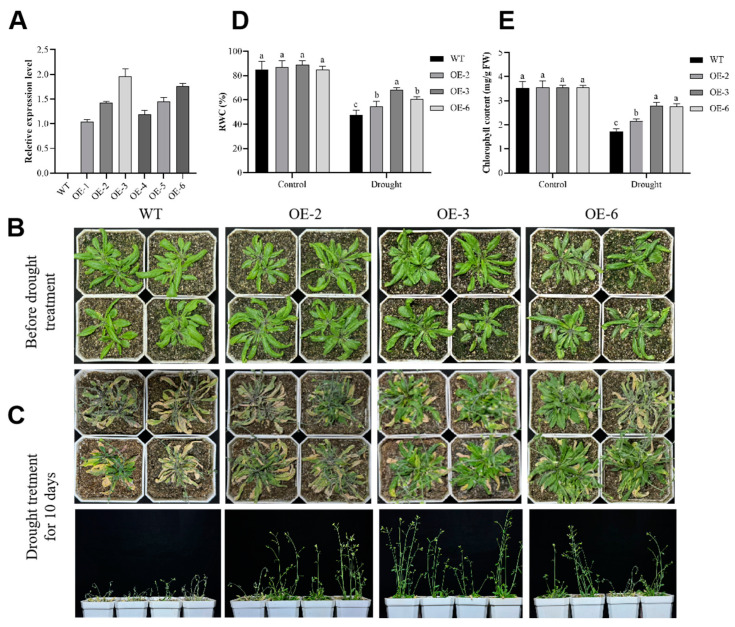
Performance assessment of *GmADF13* transgenic *Arabidopsis* and wild-type (WT) plants under normal and drought stress conditions. (**A**) The relative expression levels of overexpression lines, with three biological replicates for each line. (**B**,**C**) Phenotypic observations of potted WT and transgenic plants after 10 days of growth under normal and drought stress conditions. (**D**) Relative water content (RWC) in WT and *GmADF13*-transgenic *Arabidopsis* plants under normal and drought stress conditions. (**E**) Chlorophyll content in WT and *GmADF13*-transgenic *Arabidopsis* plants under normal and drought stress conditions. Each data point represents the mean of three replicates of 80 seedlings. *AtUBQ10* served as the internal control. Data are means ± SD (n = 3). Different letters indicate statistically different groups (*p* < 0.05; ANOVA with Tukey’s multiple comparison test).

*GmADF13*, under the control of the 35S promoter, was transformed into *Arabidopsis* plants, and there were six positive overexpressing lines at the T3 generation stage. We performed a qRT-PCR analysis to assess the expression levels of the lines. The experimental results indicate that the relative expression levels of *GmADF13* in the OE-1, OE-4, and OE-5 lines are comparatively low, while they are higher in the OE-2, OE-3, and OE-6 lines, which better represent the gene’s functionality. Therefore, we have chosen to focus our subsequent research on the OE-2, OE-3, and OE-6 lines (Figure 4A).

An RT-PCR analysis showed that *GmADF13* was expressed in the OE-2, OE-3, and OE-6 lines but not in the WT plants (Supplementary Figure S1). The seeds of three transgenic and WT lines were sown in soil. After these plants had grown for 3 weeks under normal conditions, they were no longer watered (Figure 4B). After 10 days of this natural drought treatment, most of the leaves of the WT plants turned yellow, curled at the leaf tips, and were dehydrated or dead (Figure 4C). Compared with the WT plants, the transgenic plants displayed more healthy leaves and better growth, consistent with the relative water content (RWC) and chlorophyll measurement results (Figure 4D,E). In summary, the *GmADF13* gene may improve the drought resistance of the plant.

**Figure 5:**
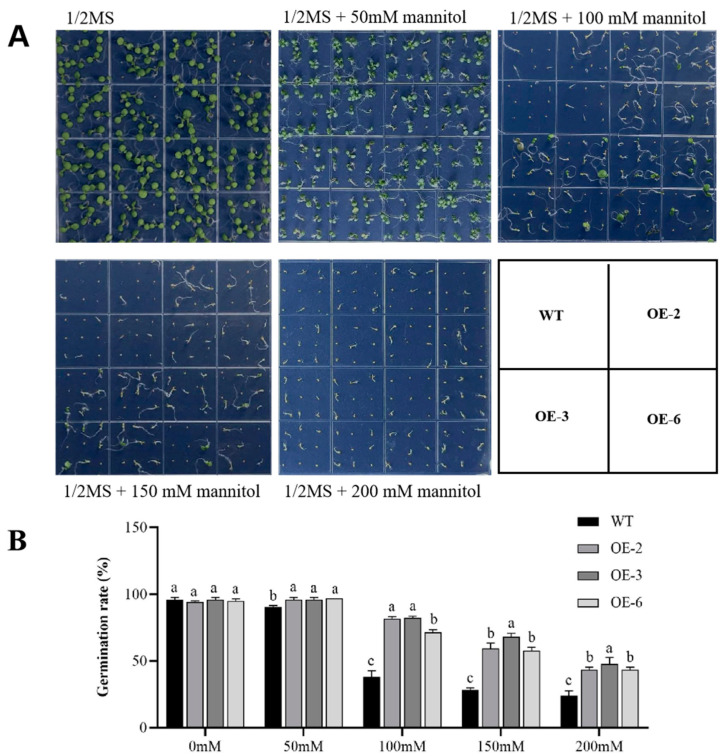
Overexpression of *GmADF13* enhanced seed germination rates under mannitol treatment. (**A**) Figurative illustration of germination percentage of WT and overexpressed lines in normal and drought conditions. (**B**) Germination percentage determination in 1/2 MS media and 1/2 MS media with 50, 100, 150, and 200 mM mannitol. Different letters indicate statistically different groups (*p* < 0.05; ANOVA with Tukey’s multiple comparison test).

In addition to the natural drought treatment, the seeds of three transgenic and WT lines were sown in one-half-strength Murashige and Skoog (1/2 MS) medium to further examine the effects of drought stress on plant development. To investigate the effect of drought stress on the *Arabidopsis* germination rate, the transgenic and WT seeds were germinated on 1/2 MS medium containing various concentrations of mannitol (Figure 5A). After a week of growth, there was no significant difference in the growth and morphology of the transgenic and WT plants without mannitol (Figure 5B), suggesting that the overexpression of *GmADF13* does not affect plant growth and development under normal conditions. Although the germination rate of transgenic and WT plants decreased as the mannitol concentration increased, the germination rate of the transgenic plants was always higher than that of the WT plants (Figure 5B). In 1/2 MS medium containing 200 mM mannitol, the three transgenic lines showed seed germination rates of 43.4%, 47.9%, and 42.4%, respectively, whereas the WT seeds only had a 24% germination rate within 7 days.

**Figure 6:**
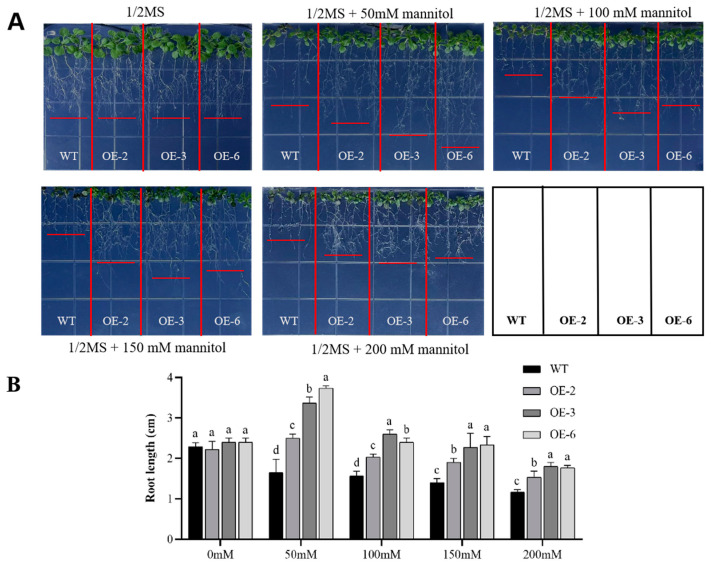
Overexpression of *GmADF13* enhanced root length under mannitol treatment. (**A**) Figurative illustration of root length of WT and overexpressed lines in normal and drought conditions. (**B**) Root length determination in 1/2 MS media and 1/2 MS media with 50, 100, 150, and 200 mM mannitol. Different letters indicate statistically different groups (*p* < 0.05; ANOVA with Tukey’s multiple comparison test).

Drought stress treatments significantly impact plant root growth [49]. In the 1/2 MS medium without mannitol, the growth of the transgenic lines was generally similar to that of the WT plants, with no apparent change in root growth (Figure 6A), suggesting that the overexpression of *GmADF13* does not affect the growth of plant roots under normal conditions. Under mannitol treatment conditions, the growth of transgenic plants and WT plants was inhibited to varying degrees. The plant rosette leaves became smaller, and the root lengths became shorter as the mannitol concentration increased (Figure 6A). However, compared with the WT plants, the transgenic lines developed larger rosette leaves and longer roots at all concentrations (50, 100, and 200 mM) (Figure 6). Interestingly, the roots of the transgenic plants were the longest under the 50 mM mannitol treatment.

### 2.4. GmADF13 Improved Drought Tolerance in Transgenic Soybean Hairy Roots

**Figure 7:**
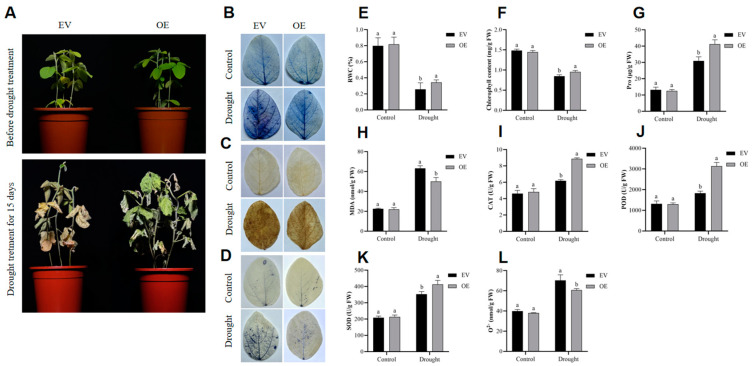
Phenotype and physiological analysis of *GmADF13* transgenic soybean hairy roots under drought stress. (**A**) Phenotypes of EV and OE transgenic soybean plants treated under drought stress for 15 days. Trypan blue staining (**B**), DAB staining (**C**), NBT staining (**D**), relative water content (RWC) (**E**), chlorophyll content (**F**), proline (Pro) content (**G**), malondialdehyde (MDA) content (**H**), catalase (CAT) content (**I**), peroxidase (POD) content (**J**), superoxide dismutase (SOD) content (**K**), and superoxide anion (O^2−^) content (**L**) of the leaves of EV and OE plants grown under drought treatment or normal control conditions for 15 days. Data are means ± SD (n = 3). Different letters indicate statistically different groups (*p* < 0.05; ANOVA with Tukey’s multiple comparison test).

To analyze the relationship between *GmADF13* and the drought stress response in soybean seedlings further, we generated two transgenic hairy root composite soybeans, OE and EV (the OE plants were transformed with *GmADF13* and the EV plants had an empty vector, respectively). The overexpression of *GmADF13* improved their resistance to drought stress (Figure 7). Plants with hairy roots emitting red fluorescence under a green excitation light were successfully transformed (Supplementary Figure S2). A qRT-PCR analysis revealed a significantly higher transcription level of *GmADF13* in the hairy roots of the OE soybean seedlings compared to those in the EV seedlings under normal conditions (Supplementary Figure S3). 

The EV plants had similar phenotypes to those of the OE plants before the drought treatment. After 15 days of water deprivation, the EV plants exhibited severe water loss, significant withering, and defoliation (Figure 7A). The results showed that the RWC (Figure 7E) and chlorophyll (Figure 7F) levels of the OE leaves were higher than those of the EV plants after drought stress, indicating that the OE plants suffered less damage and showed an advantage in terms of growth compared to the EV plants. 

To investigate the underlying physiological mechanisms of *GmADF13* on plant stress tolerance, the proline (Pro), malondialdehyde (MDA), catalase (CAT), peroxidase (POD), superoxide dismutase (SOD), and superoxide anion (O^2−^) levels in the EV and OE plants were measured under normal and drought stress conditions. These factors in the OE plants did not differ from those in the EV plants under normal conditions. However, the OE plants experienced delayed leaf wilt (Figure 7A). The Pro (Figure 7G), CAT (Figure 7I), POD (Figure 7J), and SOD (Figure 7K) levels in the OE plants were higher than those in the EV plants, and the MDA (Figure 7H) and O^2−^ (Figure 7L) levels were lower than those of the EV plants under drought stress.

Trypan blue staining can help visualize the degree of damage to leaves as dead cells can be stained, but living cells cannot. DAB (3,30-diaminobenzidine) and NBT (nitroblue tetrazolium) staining reveal the accumulation of harmful products in leaves. DAB staining detects the hydrogen peroxide (H_2_O_2_) level, while NBT staining detects O^2−^ in plant leaves. We used trypan blue (Figure 7B), DAB (Figure 7C), and NBT (Figure 7D) staining to measure the cell viability of the EV and OE plant leaves under drought stress. No significant difference was observed between the OE and EV plants under normal growth conditions. However, following drought treatments, the OE leaves exhibited a significantly lower color depth compared to that of the EV plants across all three staining methods, indicating less damage to the OE plants under drought stress. These findings suggest a positive regulatory role of *GmADF13* in drought stress tolerance in transgenic hairy root composite soybeans.

### 2.5. GmADF13-OE Plants Exhibit Increased Drought-Inducible Gene Transcription

**Figure 8:**
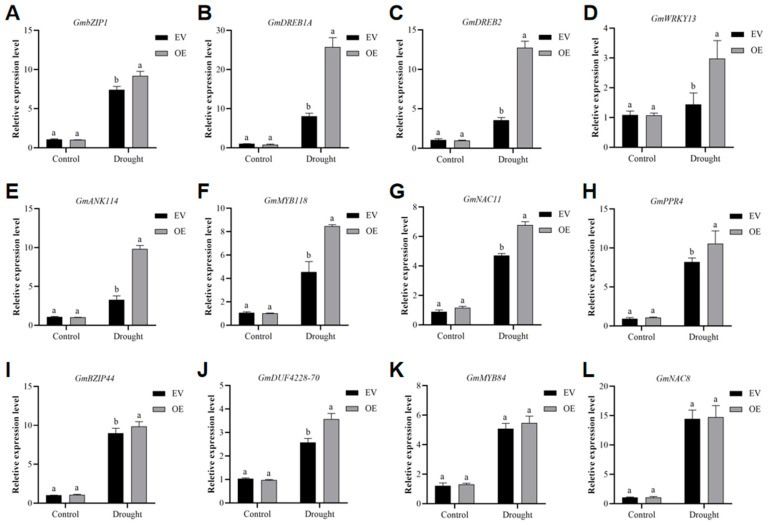
The expression levels of drought-stress-related genes *GmbZIP1* (**A**), *GmDREB1A* (**B**), *GmDREB2* (**C**), *GmWRKY13* (**D**), *GmANK114* (**E**), *GmMYB118* (**F**), *GmNAC11* (**G**), *GmPPR4* (**H**), *GmbZIP44* (**I**), *GmDUF4228-70* (**J**), *GmMYB84* (**K**), and *GmNAC8* (**L**) based on qRT-PCR. The tubulin gene was used as an internal control. Data are means ± SD (n = 3). Different letters indicate statistically different groups (*p* < 0.05; ANOVA with Tukey’s multiple comparison test).

To further analyze the molecular mechanisms underlying *GmADF13*-mediated responses to drought stress, we selected 12 genes known to respond to drought stress based on prior reports [45,50,51,52,53,54,55,56,57,58,59,60] (Figure 8). A comparative analysis of the transcript levels of several drought-inducible genes between the OE and EV plants under normal and drought conditions revealed no significant differences (Figure 8). However, under the drought treatment, ten genes exhibited differential expressions between the OE and EV plants: *GmbZIP1* (Figure 8A), *GmDREB1A* (Figure 8B), *GmDREB2* (Figure 8C), *GmWRKY13* (Figure 8D), and *GmANK114* (Figure 8E). In addition, *GmMYB118* (Figure 8F), *GmNAC11* (Figure 8G), *GmPPR4* (Figure 8H), *GmbZIP44* (Figure 8I), and *GmDUF4228-70* (Figure 8J) were upregulated to some extent. There was no apparent difference in *GmMYB84* (Figure 8K) and *GmNAC8* (Figure 8L) expression with the drought treatment between the OE and EV plants. Under the drought treatment, the increased transcription levels of these genes varied, indicating that *GmADF13* may regulate some of these genes. Further studies are necessary to identify the relationships between *GmADF13* and stress-related genes in soybeans.

## 3. Discussion

Plants are vulnerable to abiotic stresses, the most notable of which is drought stress [1]. Drought can ultimately lead to a crop yield reduction through a progression of complex physio-biochemical and metabolic processes at the genetic and molecular levels [40,61]. To cope with drought stress, plants have evolved various adaptations, including drought escape, drought avoidance, drought tolerance, compatible solute accumulation, antioxidant regulation, and hormonal regulation [40,62]. When plants encounter stress, their physiological and biochemical states change to differing degrees to improve their chances of survival under stress [63]. Soybean, an important leguminous crop, is widely cultivated globally [38]. Due to its rich nutritional content, foods processed from soybeans have become one of the most favored sources of sustenance [64]. Despite the annual increase in soybean production, environmental stress remains a significant factor affecting soybean yield [6]. Among various environmental stresses, drought is one of the most impactful abiotic stressors on soybean quality [5]. Previous studies have shown that drought stress reduces soybean germination rates and net photosynthetic rates, leading to significant decreases in biomass accumulation, pod set rates, and per plant grain yield [65,66]. Given its importance as a staple food crop, research on soybean drought resistance has garnered extensive attention [6].

ADF functions in various abiotic stress processes in plants [26]. Our research shows that GmADF13 is a typical plant ADF protein that has a highly exploited actin-binding module: the ADF-H domain [67], which cuts or depolymerizes F-actin, thus participating in plant growth and development as well as the stress response (Figure 1). A phylogenetic tree analysis showed that the GmADF13 protein formed a large branch with the CcADF5, AtADF5, and ZmADF5 proteins, indicating that these have a close evolutionary relationship (Figure 2). Additionally, *AtADF5* and *ZmADF5* have been reported to participate in plant response abiotic stresses [35,36], suggesting that *GmADF13* may have similar functions.

*GmADF13* was drastically upregulated under drought stress conditions, indicating that it might play a vital role in plant response to abiotic stress [48]. However, the function of *GmADF13* in stress tolerance remains unknown, requiring further investigation. In this study, we cloned the *GmADF13* gene from soybeans and obtained transgenic *Arabidopsis* and hairy root composite soybeans to investigate its potential function. The results showed that the overexpression of *GmADF13* enhanced the drought survivability of the transgenic lines. Specifically, the transgenic *Arabidopsis* grew better than the WT plants after a drought, and the germination percentage and root length of the *GmADF13* overexpressed lines were higher than those of the WT under different mannitol concentrations (Figure 4, Figure 5 and Figure 6). Drought treatment caused apparent differences in growth between thee soybean OE and EV lines. Following 15 days of drought treatment, all of the hairy root plants exhibited a gradual yellowing and wilting of their leaves, with the EV control displaying a higher level of sensitivity compared to that of the OE plants (Figure 7A).

Roots serve as the primary organ for water and mineral nutrient absorption in plants, playing a pivotal role in sensing and adapting to various abiotic stresses [49,68]. The root system perceives drought stress, which transmits a signal to the leaves through a feedback mechanism, causing the leaves to be damaged to varying degrees. Our results showed that the *GmADF13* overexpression in soybean hair roots reduced water loss and chlorophyll degradation while improving the viability of the plants under drought stresses (Figure 7E,F). Pro is an important indicator used to measure the effects of abiotic stress on plant growth [65,66]. As a protective agent against drought stress, Pro assists in the osmotic adjustments that maintain cell turgor, which prevents water loss from cells and plays a vital role in plant protection [69]. Under drought stress, the transgenic lines possessed higher pro content compared to the WT, suggesting that their cytoplasm may be more stable and more effective in preventing dehydration damage, indicating that *GmADF13* confers a tolerance to drought stress to the soybean (Figure 7G). 

Abiotic stress leads to ROS accumulation, which damages the cell membrane, lipids, carbohydrates, and protein structure, affecting plant growth and development [70]. O^2−^ and H_2_O_2_ are the primary sources of ROS, and they can lead to the oxidative destruction of cells. The O^2−^ level in the leaves of the OE plants was significantly lower than that in the controls, and the results of NBT staining were also the same as those for these indicators (Figure 7L). Additionally, the DAB staining results showed that the EV plants accumulated more H_2_O_2_ compared to the OE plants. This indicates that *GmADF13* can enhance plant drought resistance by reducing the accumulation of ROS in cells. Thus, *GmADF13* is involved in the abiotic stress response and enhances plant tolerance to drought stress.

POD and SOD serve as vital antioxidants, playing a pivotal role in safeguarding plants against various stressors [71]. They are effective in mitigating the accumulation of ROS in plants when subjected to stress [72]. Additionally, the enzyme CAT functions as a key osmotic regulator, helping to reduce excess ROS levels in plants [73]. As such, the activities of POD, SOD, and CAT are crucial parameters influencing the plant’s response to drought stress. Moreover, MDA, a byproduct of enzymatic and oxygen radical-induced lipid peroxidation, serves as an endogenous marker of genotoxicity. Elevated MDA levels often indicate the extent of membrane damage [74]. When plant tissue enzymatic and membrane systems are compromised, MDA levels tend to rise significantly [75], making it a valuable indicator of plant resilience to external stresses. This study assessed the physiological and biochemical parameters of transgenic soybeans subjected to drought stress. Notably, no substantial differences were observed between the OE and EV plants under normal conditions (Figure 7H–K). However, under drought stress conditions, the activities of POD, SOD, and CAT were markedly higher in the OE plants compared to those in the EV plants, whereas the MDA content exhibited the opposite trend (Figure 7H–K).

Plants have developed flexible molecular and cellular mechanisms to fight against various abiotic stresses. The overexpression of *GmbZIP1* probably regulates stomatal closure and reduces water loss, consequently enhancing drought stress tolerance [51]. *GmDREB1A* can be used to improve the drought tolerance of important crops by gene transfer [52]. Similarly, *GmDREB2* functions as an important transcriptional activator; the overexpression of *GmDREB2* activates the expression of some downstream genes involving free proline biosynthesis, enhancing the drought stress tolerance in transgenic plants [53]. Studies have shown that *GmWRKY13* may function in both lateral root development and the abiotic stress response [54]. Moreover, *GmANK114*, *GmMYB118*, *GmNAC11*, *GmPPR4*, *GmbZIP44*, and *GmDUF4228-70* are also involved in stress responses. Our results indicate that following drought stress, the expression levels of the selected genes in OE plants are upregulated compared to the EV control, especially *GmbZIP1* (Figure 8A), *GmDREB1A* (Figure 8B), *GmDREB2* (Figure 8C), *GmWRKY13* (Figure 8D), and *GmANK114* (Figure 8E), which were significantly upregulated. This result suggests that *GmADF13* responds to stress by changing the expression of drought-related genes. However, it is not clear how *GmADF13* affects the function of other genes to enhance stress resistance in soybeans. Furthermore, the transgenic soybean hairy roots generated through *Agrobacterium rhizogenes*-mediated hairy root transformation represent a transient transformation method that does not ensure stable inheritance. As such, it remains unclear whether overexpressing *GmADF13* can enhance soybean yield under drought stress, and further investigations with stable genetically modified soybean plants are warranted to confirm this.

## 4. Materials and Methods

### 4.1. GmADF13-OE Plants Exhibit Increased Drought-Inducible Gene Transcription

*Arabidopsis* (Columbia-0, Col-0), soybean (Williams 82, W82), and tobacco used in the present study were preserved as germplasm resources at the School of Agricultural Science and Engineering of Liaocheng University, China.

Seeds of transgenic and WT *Arabidopsis* were incubated with 1 mL of 75% (*v*/*v*) ethanol for 5 min and 3% (*w*/*v*) sodium hypochlorite solution for 10 min. Then, they were washed five times with sterile water. The surface-sterilized seeds were evenly distributed in solid 1/2 MS medium (pH 5.8) containing 2% (*w*/*v*) sucrose and 1% (*w*/*v*) agar. The seeds were then vernalized for 3 d, stored in the dark at 4 °C, and maintained in a culture room at 22 °C, under 16 h light/8 h dark cycle, and with 60% relative humidity. T0–T3 (transgenic generation 0–3) seeds were screened, and T3 progeny were used for the final treatment. The seeds of soybean W82 were soaked in water, germinated for 6 h, planted in a pot, and grown under a 16 h light/8 h dark photoperiod and a 25 °C/18 °C (day/night) cycle.

### 4.2. GmADF13 Cloning, RNA Extraction, and qRT-PCR

For *GmADF13* cloning, RNA was extracted from young soybean sprouts. According to the manufacturer’s instructions, total RNA was extracted with the E.Z.N.A. Plant RNA Kit (No. R6827-01; Omega Bio-Tek, Norcross, GA, USA). The integrity and quality of the extracted RNA were measured by gel electrophoresis and NanoDrop TM Lite Spectrophotometer (No. ND-LITE-PR; Thermo Scientific, Waltham, MA, USA). Reverse transcription of RNA into complementary DNA (cDNA) was performed using the HiScriptIII RT SuperMix for PCR (No. R312-02; Vazyme Biotech, Nanjing, China). Specific amplification primers were designed for the target gene using the online tool Primer3 (https://github.com/primer3-org (accessed on 6 January 2024)), and PCR amplification was conducted using High-Fidelity Enzyme 2×T8 High-Fidelity Master Mix (No. TSE111; Tsingke Biotechnology, Beijing, China) and cDNA as the template. The PCR conditions were 34 cycles of 95 °C for 15 s, 58 °C for 15 s, and 72 °C for 30 s. The PCR product was purified with the FastPure Gel DNA Extraction Mini Kit (No. DC301-01; Vazyme Biotech). All materials were stored at −20 °C until use. The RT-PCR primers are listed in Supplementary Table S1.

For qPCR, RNA was extracted according to the method above and then reverse-transcribed into cDNA with HiScriptⅢ RT SuperMix for qPCR (+gDNA wiper) (No. R323-01; Vazyme Biotech). The qRT-PCR analysis was performed using SYBR Premix Ex TaqTMⅡ (No. DRR041A; TaKaRa Bio, Kusatsu, Shiga, Japan). The qRT-PCR conditions were 95 °C for 30 s followed by 34 cycles of 95 °C for 30 s, 60 °C for 30 s, and 95 °C for 15 s. The RNA transcript fold changes were calculated using the 2^−△△Ct^ method [76]. The *tubulin* gene (*Glyma.05G157300*) was the internal control for soybeans, and *UBQ10* (*At4g05320*) was the internal control for *Arabidopsis* [77,78]. The qRT-PCR primers are listed in Supplementary Table S1.

### 4.3. Phylogenetic Analysis

The amino acid sequences of different plant species were obtained from the NCBI database (https://www.ncbi.nlm.nih.gov (accessed on 6 January 2024)). The alignment of multiple sequences of proteins from different plant species was performed using DNAMAN 9.0 (https://www.lynnon.com/dnaman.html (accessed on 6 January 2024)) and the “multiple sequence alignment” function with default parameters. Using MEGA 11.0 (https://megasoftware.net/ (accessed on 6 January 2024)) software, a phylogenetic tree was constructed using the neighbor-joining method with the following parameters: Poisson model, pairwise deletion, and 1000 bootstrap replications.

### 4.4. Subcellular Localization of GmADF13

The CDS of *GmADF13* was amplified using gene-specific primers and homologous recombination technology, without a termination codon. The purified PCR product was fused with the upstream *GFP* gene, generating a GmADF13-GFP fusion vector driven by the CaMV 35S promoter, with the 35S::GFP vector used as a control. Subcellular localization experiments were conducted by Wuhan BioRun Biosciences Co., Ltd. (Wuhan, China).

Transfer the constructed vector plasmid into *Agrobacterium tumefaciens* (GV3101). Resuspend the bacteria in 10mM MgCl_2_ solution (containing 120 μM AS) and adjust the OD600 to approximately 0.6. Inject the cell solution, using a 1mL syringe, into the lower epidermis of tobacco leaves with the apical meristem removed, making sure to label each injection site. Culture the injected tobacco plants under low light conditions for 2 days, then take the labeled tobacco leaves to prepare them for slides. Observe the slides under confocal laser scanning microscope (No. LSM980; Carl Zeiss, Oberkochen, Germany) and take pictures. 

The protoplasts were transiently transformed by adding 20 µL vector (10 µL GmADF13-GFP and 10 µL Marker-GFP) to a 200 µL protoplast solution, which was then gently mixed and incubated at room temperature for 12 h. Green fluorescence was detected by confocal laser scanning microscopy (No. LSM980; Carl Zeiss) and pictures were taken.

### 4.5. Generation of Transgenic Arabidopsis Plants Overexpressing the GmADF13 Gene and Screening

To transform *Arabidopsis*, the *GmADF13* CDS was cloned into the pCAMBIA1305 vector using MonClone Single Assembly Cloning Mix (No. 250548; Monad Biotech, Wuhan, China) and was designated pCAMBIA1305.1-ADF13. Sangon Biotech (Shanghai, China) was commissioned to verify the accuracy of the sequence using Sanger sequencing, and heterologous transformation into *Arabidopsis* was performed using the floral dipping method [79]. T1-generation seeds were collected from T0-generation *Arabidopsis* seedlings and sown on kanamycin media to select the segregant lines. T3 stably transformed homozygous lines were used to evaluate drought tolerance. 

### 4.6. Agrobacterium Rhizogenes-Mediated Transformation of Soybean Hairy Roots

Transgenic hairy root composite soybean plants were constructed using the method described by Teng et al. (2023) [80]. Briefly, W82 seeds were washed with 75% ethanol for 3 min and rinsed in sterile water five times. Three seeds were placed in each pot, and three pots formed a group. When the seedlings had just sprouted, the cotyledon unfolded, and the first leaf had not yet appeared, *Agrobacterium rhizogenes* K599 harboring the overexpression vector and empty vector were transformed into the OE and EV plants. Fourteen days later, soybean plants with hairy roots were selected, and the plants verified by green excitation light at 540 nm, emission at 600 nm, and PCR were used in this study.

### 4.7. Drought Stress Assays of Transgenic Arabidopsis and Hairy Root Composite Soybeans

For germination analysis, seeds were sown on 1/2 MS growth media supplemented with various concentrations of mannitol (50, 100, 150, and 200 mM) (No. A600335; Sangon Biotech). After 3 days of vernalization at 4 °C, the seeds were transferred to standard conditions for germination. The seeds were considered germinated when radicles emerged from the seed coats. The germination of each line in one week was observed and recorded, and the germination rate was determined by dividing the number of germinated seeds by the total number of seeds. At least 80 seeds per genotype were measured.

For root growth analysis, sterilized WT and T3 transgenic lines seeds were sown on 1/2 MS growth media, and seedlings of the same length were selected and transferred to growth media containing different concentrations of mannitol (50, 100, 150, and 200 mM) (No. A600335; Sangon Biotech). After one week of growth, the plate was placed vertically in the incubator so the roots grew downwards toward the ground. At least 80 seedlings per genotype were measured. 

To test drought tolerance at later developmental stages, the seeds of WT and T3 transgenic *Arabidopsis* lines were sown and grown for three weeks in a pot with a 3:1 mixture of vermiculite and soil, watered regularly, and then subjected to drought stress by withholding water for 10 days. Then, plant phenotypes were observed. At least 80 seedlings were measured in each line. 

After two weeks of drought treatment, leaves with the same leaf position were selected for staining and physiological index determination for soybean hairy root composite plants. RNA extracted from transgenic hairy roots was isolated to detect the relative expression levels of related stress resistance genes. All the experiments were conducted in triplicate.

### 4.8. Relative Water and Chlorophyll Content

The chlorophyll content can reflect the strength of photosynthesis, and the RWC can be used as an indicator of plant water status [81,82]. Both values can be used to quantify the effects of drought stress on plant development. Fresh leaves of EV and OE plants in the same leaf position were selected for weighing to determine the RWC. Specifically, the leaves were cut and quickly weighed to obtain the fresh weight of the leaves. The leaves were completely immersed in sterile water for 12 h, carefully removed, drained of any excess water on the surface of the blade using absorbent paper, and weighed to obtain a saturated weight. Finally, the leaves were wrapped with tin foil and placed in a constant-temperature oven at 105 °C for 15 min, then baked in a constant-temperature oven at 65 °C until constant weight, and weighed to obtain the dry weight (No. ME204E; Mettler Toledo, Columbus, OH, USA). To determine the chlorophyll content of the leaves, fresh leaves from the EV and OE plants in the same leaf position were selected and evenly cut into strips, immediately immersed in a mixture of 50% anhydrous ethanol and 50% acetone, and incubated in a dark incubator for 12 h. Then, the absorbance values of different samples at 645 nm and 663 nm were measured with a NanoDropLite spectrophotometer (No. ND-LITE-PR; Thermo Scientific). Each measurement was performed with three biological replicates. 

### 4.9. Measurement of Proline, Malondialdehyde, Antioxidant Enzymes, and Superoxide Anion Levels

The determination of Pro, MDA, CAT, POD, SOD, and O^2-^ contents was entrusted to Nanjing Convinced-test Technology Co., Ltd. (Nanjing, China), which provided corresponding technical support. All measurements were performed with three biological replicates. 

### 4.10. NBT, DAB, and Trypan blue Staining

Leaves of the EV and OE plants at the same leaf position were picked and submerged in 0.4% trypan blue (No. C0040; Solarbio), DAB (No.SL1805; Coolaber, Beijing, China), and NBT (No. SL1806; Coolaber) staining solutions for 12 h. Finally, the stained leaves were immersed in 75% ethanol and heated in a water bath at 90 °C until the leaves were completely discolored. Images were taken with a Canon 50D (Canon, Tokyo, Japan) camera. 

### 4.11. Data Analysis

All experiments were performed in duplicate with at least three independent replicates. Values are expressed as mean ± standard deviation (SD). The data were analyzed and are expressed as graphs using GraphPad Prism 8.0 (https://www.graphpad.com/ (accessed on 6 January 2024)). One-way ANOVA with Tukey’s multiple comparison test was conducted using IBM SPSS Statistics (https://www.ibm.com/cn-zh/spss (accessed on 6 January 2024)). Different letters (a, b, c, d, e) indicate statistical differences between groups (*p* < 0.05), while the same letter indicates no significant difference.

## 5. Conclusions

In summary, this study reports the function of soybean *GmADF13* in regulating drought resistance. It may enhance drought tolerance by affecting the accumulation of osmoregulation substances, the balance of enzyme activities, and the reprogramming of stress-tolerance-related genes. Our results provide important candidate genes for plant drought tolerance breeding, help elucidate the mechanism of ADF protein involved in plants’ response to drought stresses, and provide a theoretical basis for plant environmental adaptation.

## Data Availability

The data are contained within the article and Supplementary Materials.

## Supplementary Materials

The following supporting information can be downloaded at: https://www.mdpi.com/article/10.3390/plants13121651/s1, Figure S1: RT-PCR analysis; Figure S2: Soybean hairy root transformation; Figure S3: Expression levels of *GmADF13* in OE and EV plants; Table S1: RT-PCR and qRT-PCR primer design; Table S2: Multiple sequence alignment; Table S3: Phylogenetic tree; Table S4: Information on genes related to drought tolerance.
