# Supplementary material for: Functional Characterization of the Soybean Glycine max Actin Depolymerization Factor GmADF13 for Plant Resistance to Drought Stress
Source: Plants (Basel). 2024 Jun 14;13(12):1651. doi: 10.3390/plants13121651 (PMC11207668; doi:10.3390/plants13121651)
Supplement: Supplementary file 1 [file plants-13-01651-s001.zip › Supplementary Figures.pdf]

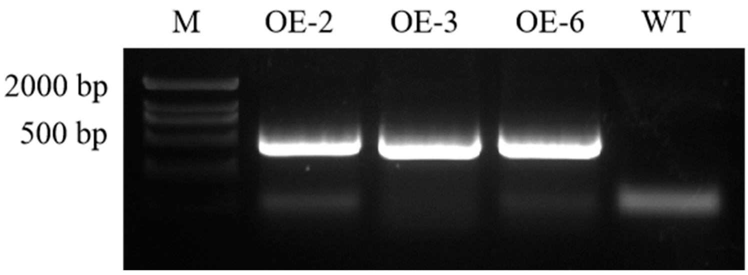

Supplementary Figure S1. RT-PCR analysis.

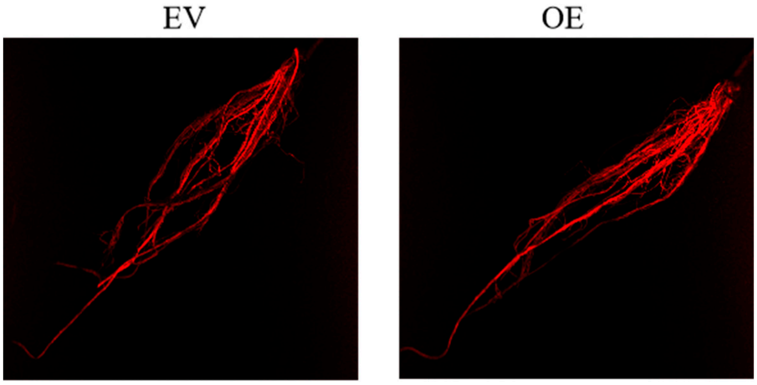

Supplementary Figure S2. Soybean hairy root transformation.

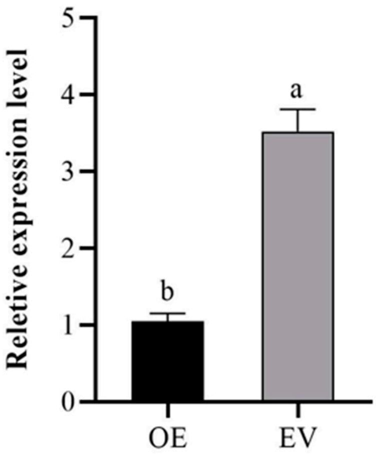

Supplementary Figure S3. Expression levels of *GmADF13* in OE and EV plants.
