# Supplementary material for: Functional Characterization of the Soybean Glycine max Actin Depolymerization Factor GmADF13 for Plant Resistance to Drought Stress
Source: Plants (Basel). 2024 Jun 14;13(12):1651. doi: 10.3390/plants13121651 (PMC11207668; doi:10.3390/plants13121651)
Supplement: Supplementary file 1 [file plants-13-01651-s001.zip › Supplementary Tables.pdf]

Supplementary Table S1. RT-PCR and qRT-PCR primer design.

Gene name	Forward primer	Reverse primer	Description
GmADF13-1	ATGGCGATGGCTTTCAAAATG	CTATTTGGCTCTGTCTTGAATTACATCG	RT-PCR
GmADF13-2	TCACCGTTGATAAGGTGGGT	CCTTGGATGTTGCGTAGAGC	qRT-PCR
GmbZIP1	GAGGTTTGGAGGGACTTG	TTGAGGATTTGATGAGCC	qRT-PCR
GmDREB1A	GTCCGCTGATTCCATTC	AAGTCGGGCTTGAGATT	qRT-PCR
GmDREB2	AGCGAAAGCAGCAGCACC	GTTAAGGCGAGCGGAAGG	qRT-PCR
GmWRKY13	AGGACACGGATACAGCAA	CATCTTCAGGGTAGGCAC	qRT-PCR
GmANK114	TATGGCCATCAGTGCATCCC	TGCCAGAATCTCATTGGCGA	qRT-PCR
GmMYB118	TTGCTTGAGATTATGGTCCTTG	CATCACCCTTTCCTTCAACC	qRT-PCR
GmNAC11	TCCCTCTTGTTCTATGCT	GTTTCCCATTATTTGCCTA	qRT-PCR
GmPPR4	TTCTTGGGGACATGAGCAGG	TCAAGGCTCCCACACTTGAC	qRT-PCR
GmbZIP44	TCGGATGCGAAAGCGTAA	TGCGTGGTGATGTCTATGGTG	qRT-PCR
GmDUF4228-70	CTTGAAAGGTTCCAATGGAAGG	CGGTGAAAACCTAACCTTGTTT	qRT-PCR
GmMYB84	GGGGAAACAGGTGGTCAA	TCTAGGCATCCAGAAACG	qRT-PCR
GmERD1	CGTCCAGAATTGCTCAACAG	TGGGGTTATAGCCTTGTTGG	qRT-PCR
Tubulin	TCTTGGACAACGAAGCCATCT	TGGTGAGGGACGAAATGATCT	qRT-PCR
UBQ10	CCTTGTATAATCCCTGATGA	AACAGGAACGGAAACATAGT	qRT-PCR

Supplementary Table S2. Multiple sequence alignment.

Species	Gene	ID	Per.ident
Vigna radiata var. radiata	*VrADF5*	*XP 014491963.1*	99.30%
Mucuna pruriens	*MpADF5*	*XP 031270817.1*	97.20%
Mangifera indica	*MiADF5*	*XP 044488578.1*	96.50%
Cicer arietinum	*CaADF5*	*XP 004505784.2*	95.10%
Trifolium pratense	*TpADF5*	*XP 045819637.1*	95.10%
Cajanus cajan	*CcADF5*	*XP 020214942.1*	95.80%
Pisum sativum	*PsADF5*	*XP 0508998151.1*	95.10%
Abrus precatorius	*ApADF5*	*XP 027355023.1*	95.10%
Vicia villosa	*VvADF5*	*XP 058730171.1*	94.41%
Medicago truncatula	*MtADF5*	*XP 013456470.1*	94.41%
Gossypium raimondii	*GrADF5*	*XP 012467545.1*	94.41%
Arachis duranensis	*AdADF5*	*XP 0159518701*	94.41%
Jatropha curcas	*JcADF5*	*XP 012081752.1*	93.01%
Diospyros lotus	*DlADF5*	*XP 052193716.1*	93.01%

Supplementary Table S3. Phylogenetic tree.

Species	Gene	ID
Arabidopsis thaliana	*AtADF1*	*OAP040781*
Arabidopsis thaliana	*AtADF4*	*NP 8512281*
Arabidopsis thaliana	*AtADF5*	*OAP07315.1*
Cajanus cajan	*CcADF2*	*XP 020202305.1*
Cajanus cajan	*CcADF4*	*XP 020212020.1*
Cajanus cajan	*CcADF5*	*XP 020214593.1*
Deschampsia antarctica	*DaADF3*	*UDB45807.1*
Oryza sativa Japonica Group	*OsADF2*	*NP 001389205.1*
Orza sativa Japonica Group	*OsADF3*	*NP 001405897.1*
Solanum lycopersicum L	*SiADF5*	*Solyc06g005360.3.1*
Triticum aestivum	*TaADF4*	*AGW22223.1*
Zea mays	*ZmADF1*	*NP 001105463.1*
Zea mays	*ZmADF3*	*NP 001358558.1*
Zea mays	*ZmADF5*	*NP 001401277.1*

Supplementary Table S4. Information on genes related to drought tolerance.

Gene	Full name	Reference
GmbZIP1	Basic domain/leucine zipper	[47]
GmbZIP44	Basic domain/leucine zipper proteins	[54]
GmDREB1A	dehydration responsive element binding	[48]
GmDREB2	dehydration responsive element binding	[49]
GmWRKY13	WRKYGQK	[50]
GmANK114	Ankyrin repeat	[46]
GmMYB84	myeloblastosis	[56]
GmMYB118	myeloblastosis	[51]
GmNAC8	NAM、ATAF1/2、CUC1/2	[41]
GmNAC11	NAM、ATAF1/2、CUC1/2	[52]
GmPPR4	Pentatricopeptide-repeat proteins	[53]
GmDUF4228-70	Domain of unknown function proteins	[55]
